# Sketches of Some of the Most Distinguished Physicians of Paris

**Published:** 1847-12

**Authors:** 


### ECLECTIC DEPARTMENT, AND SPIRIT OF THE MEDICAL PERIODICAL PRESS.

*Sketches of some of the most distinguished Physicians of Paris.*— The following sprightly portraytures are contained in an interesting series of "Notes on Medical Matters and Medical Men in Paris," communicated for the Western Journal of Medicine and Surgery. The writer is sojourning in Paris for the advantages of medical observation and improvement which that city affords. We avail ourselves in this number, as we have done before, of other interesting portions of Dr. Yandell's *notes.—Ed. Buff. Med. Jour.*

*M. Chomel*, professor of clinical medicine at the Hotel Dieu, is in his sixtieth year, though I think if it were not for a stoop in his shoulders he would not appear so old. The expression of his face is pleasant and determined. His head is round, and not badly proportioned; his eyes are black, shaded by handsome eyebrows; his nose turned up, his mouth small and round, his hair thick and gray. In manner he is considerate to his pupils, kind and conciliatory to his patients. Like the majority of French lecturers he speaks fluently, rapidly and distinctly. Making usually but few clinical remarks at the bedside, his service cannot be considered interesting, unless his lectures—which during the winter he delivers three times a week in one of the amphitheatres of the hospital—be considered as forming a part of it, when it is at least not wholly devoid of interest, if lacking in that spirit which makes the service of some of his colleagues so attractive.

*M. Rostan*, also professor of clinical medicine at the Hotel Dieu, I should say was near the same age as M. Chomel, though he is a younger looking man, of medium height, round body, broad, short, open and agreeable face, good head, medium forehead, thin, grayish hair wanting on top, hazel eyes, small mouth and common nose. This portrait may not strike you as being very like the engravings that we see of the great American philosopher; but I never was more forcibly impressed with the resemblance between any two faces than when, coming one morning from the Hotel Dieu where I had seen M. Rostan, I saw an engraving of Dr. Franklin. Rostan is a pleasing and occasionally an animated speaker, clear in his reasoning, and careful in his deductions, and always exhibiting in his remarks, both at the bedside and in the lecture room, the warmest desire to communicate sound, valuable and practical knowledge to the troop of students, native and foreign, who attend his clinics.

*M. Andral* is a very ordinary looking man, fifty years of age five feet eight or ten inches in height. His hair is grayish, thin on the crown of a medium sized head, long behind and in front. With a stoop in his carriage, a slim and not very well formed person, a large and long nose, and thick lips, no one would ever point him out as the professor of pathology and general therapeutics in the *Ecole de Medicine.* His service at *La Charite* is attended by few, in fact by no students, because he does not seem to care about imparting instructions at the bedside, As a lecturer I spoke of him in a former letter.

*M. Bouillaud,* professor of clinical medicine at La Charite, in many respects is Andral's opposite. His carriage is erect, his head unusually fine, his forehead broad, round, high and full, his eyes dark and animated, overhung by black, thick eyebrows; his hair thin, long, and black, sprinkled here and there with gray; for he, too, is fifty years old. His face is thin and his cheek bones high, his nose large and one of the most expressive features of his countenance. Intellectuality and intensity are the predominant and indelible characteristics of the man. In his bearing towards his patients he is unnecessarily severe and abrupt, and towards his students touchy and ill-tempered. He lectures with great earnestness, fire and grace. His sentences are now round, beautifully turned, ringing and compact, and again, as he becomes engaged in the discussion of his favorite doctrines, or even when presenting some important question, his thoughts flowing as rapidly as lightning, and growing more earnest as he proceeds, they become nervous, and sometimes abrupt, and almost jerking.

*M. Louis,* one of the physicians at the Hotel Dieu, is a striking looking, finely formed man, standing six feet in his socks, sixty years of age, with a fair head, high forehead, thin, short, brown hair, broad, thick whiskers, straight, handsome nose, crossed by a pair of spectacles, well shaped mouth, round chin, and blue eyes. He struck me as being a fine looking, sour-tempered man, particularly unkind to his patients, and not especially pleasant to the three or four pupils who follow him.

Of *M. Rayer,* author of an able work on cutaneous diseases, I have already spoken.

*M. Cruveilhier* is a round, pleasing, amiable looking man, fifty-six or fifty-seven years old, five feet seven or eight inches in height, with regular, handsome features, eyes dark and large, mouth expressive of amiability, hair gray, and small whiskers of corresponding color. His manner to his patients is kind. As a lecturer he ranks low, probably lower than any other professor in the School of Medicine· His voice is sadly against him, being weak and without either volume or music. He speaks without animation, in a sing-song, and dwells long upon what is frequently least important in his subject, often seeming not to seize—as he certainly in many instances fails in developing—the cardinal points of his matter.

Very few students attend his lectures; and when they have been, as I may say, spoiled by listening to such speakers as Denonvilliers, Dumas, Trousseau, and others of the same order, I only wonder that any at all have the patience to *endure* him through an hour. I say endure him, because one must have good ears, keep them well open, and sit close to him, in order to hear the fifth part of what he says.

*M. Trousseau* is an elegant looking, and very handsome man, of fair complexion, long, black hair, which he combs directly back from his face, long Bourbon nose, blue eyes somewhat sunk in his head, which is of medium size. He wears broad thick, half-whiskers. He is an orator. Clear, earnest and graceful, with a fine voice, person and eye, he at first commands the attention of his auditory, and then fixes it by the attractive manner in which he presents the well digested matter of his numerously attended and instructive lectures. M. Trousseau is not yet quite forty-seven years of age, and is, as you know, professor of therapeutics and materia medica in the School of Medicine.

*M. Piorry*, professor of internal pathology in the School of Medicine, is about fifty-three years old, He is a tall, round-shouldered man, with a long face, long nose, wide mouth, thin lips, small black eyes, and immense black whiskers. He speaks well, that is to say as well as a man without grace of manner or person, or flexibility of voice, can speak. His style is earnest, and could he throw a little grace into his gesticulations, and give the least possible softness to his voice, he would please much more than he does. The expression of his face, like the tones of his voice, is *hard*, and not intellectual, but firm, unbending and determined—in a word, indicative of the character of the man. Laborious and persevering, no task and no number of defeats deter him from the pursuit of his hopes, He said, while a young and unknown physician, "I will one day fill a chair in the School of Medicine." Five unsuccessful *concours*, so far from disheartening him, seemed to inspire him with new and greater determination, and at the sixth, triumphing over every obstacle, he demonstrated in too unmistakeable terms his capacity, and was raised to the chair which he now occupies. His eye is sharp and penetrating, and with his head slightly sunk between his broad shoulders, his huge whiskers, and his stony voice, he strikes one, after all—at least he did me—as being by nature better fitted for a commander of infantry than a teacher of medicine.

*M. Dubois*, professor in the faculty, (*Clinique d' Accouchement a l' Hopital de la Faculte*,) is about the age of M. Piorry. He is not so tall, and is the mildest, most amiable, benevolent and harmless looking man imaginable. His complexion is fresh; his hair, wanting on the crown, is brown, and thin on the sides; his nose is long, his chin long and round, and his eyes blue. There is a singular depression about the center of his head, seeming as if produced by some sharp-edged heavy body, which had pressed gradually upon the middle of the parietal bones and raised up the frontal and posterior portion of the cranium. As a lecturer, he is pleasant, popular and practical.

These are unfinished and defective portraits—copied from my note-book—of some of the principal Parisian physicians. In all cases I have given my opinions candidly, and my impressions fully. I have spoken, and will only speak of the different gentlemen as they appeared to me in the wards and amphitheatres of the hospitals, and in the halls of the School of Medicine. And now, after having, with probably as unprejudiced a mind as one could possess, followed in their visits and attended some of the lectures of the greater part of the men to whom I have allued, my opinion is, that Bouillaud, of La Charite, is the greatest man of them all; and if to his other qualities he joined the kindliness of manner to the patients entrusted to his care, possessed by Dr. Norris, of Philadelphia, I should say that he was my choice of all the physicians I have ever seen. I do not here speak of his doctrines, as I do not intend speaking of those of any of the others; but I wish to be understood as alluding especially to his powers as a diagnostician.

Chomel seems to me to belong to the bygone generation. He is at best but an ordinary man, and owes the professional position which he occupies as much to his kindliness of manner to his patients and his pupils, aided by other circumstances, as to any particular strength of mind or fund of knowledge.

Rostan as a teacher at the bed side, is without an equal in Paris; but he has occasionally wandered from the austere and rugged paths of science, and worshipped at other than her altars, and, as is inevitably the consequences, except in men of the hghest order of mind —and in some cases even with them—although endowed with a fair share of talents, and of very popular manner, he has halted this side the great goal, and will never be canonized.

Andral belongs to no common order of men; but there is ever an uncertainty in his mind as to his diagnosis—a laboring after something which he does not reach—a timidity in the formation of his opinions, and a hesitancy in their expression, which deters one from forming that estimate of him which he might otherwise be disposed to do.

Cruveilhier, while considerate to his patients, is a better anatomist than physician.

Rayer, with all his cumbrousness of person, is unquestionably the possessor of a clear, far-seeing, bold and discriminating mind. His wards usually abound in interesting cases, but from his making so few remarks his visits are comparatively profitless to the student.

Louis is not the great man in Paris that he is in the United States; but divesting the mind of the opinion entertained of him in either place, is he a great physician? I answer unhesitatingly in the negative, to this question. As we all have our own ideas of a good lecturer, so we have them of a great physician. To be a great physician, a man must only be able to arrive at a diagnosis, but must be able to do so quickly; he must not only be able to ask questions of the patient which will put him upon the desired track, but he must do it, and not confound the person with a long, tedious and useless catechising; and above all things else, his diagnosis made, and his prognosis given, the autopsy—if the disease terminate fatally—must support the one, and the result of the case confirm the other. Louis can make a diagnosis, but it is always after a long examination of the disease, and useless questions to the patient; and too often, as any one who has followed him in his visits can testify, has his diagnosis been contradicted at the autopsy, which, at last, in most cases, is the only true and legitimate method of ascertaining the capacities of the physician, and the accuracy and value of his knowledge.

I look upon Andral as superior to Louis; Rayer as superior to Andral; and Bouillaud as superior to them all. There is a cast about the mind of Bouillaud which in our eyes is to be preferred to that of any of the others. Bold, vigorous, quick and acute, he is endowed with perceptive powers to an unusual extent, Original and well disciplined, he generalizes and classifies with rapidly and correctness; discriminating, he readily detects errors, and, fearless, he independently exposes it. His powers of generalization, backed by a strong disposition to exercise them, have undoubtedly led him to advance principles which many of his brethren deem untenable. The quickness of his perception conveys him with astonishing celerity to the core of a subject, and he is vexed because others, less fortunate in his particular endowment, still traverse with slow and measured steps what he has passed at a single bound. Bouillaud's diagnosis made, he reveals it on the spot. Louis, as a general rule, although doubtless in the majority of cases he has arrived in his own mind at the diagnosis of the disease, does not avow it, and it is sometimes four or five days before he allows it to leak out. It would be folly to question Louis' knowledge of the physical signs of disease, but the want of powers of combination, which we suppose no unprejudiced man will claim for him, cripples him in his deduction, biases him in his opinions, creates distrust of them in his own mind, and inspires an unpleasant doubt of their correctness in the minds of those who follow him in his visits. Bouillaud, it may be said, is over-confident. The rapidity with which he diagnoses would at first lead one to this opinion; but then, if one will take the trouble to eliminate gradually what he has probably induced on the instant, he will find in the majority of instances that Bouillaud has reason to be confident, because he has arrived at the truth. Bouillaud is irritable—he belongs to the *genius irritabile;* Louis is irritable, without being able to establish any claims to membership in the same class.—*Notes on Medical matters in Paris, in Western Journal of Medicine and Surgery.*

